# Delayed cord clamping during elective cesarean deliveries: results of a pilot safety trial

**DOI:** 10.1186/s40748-018-0083-3

**Published:** 2018-07-04

**Authors:** Caroline J. Chantry, Aubrey Blanton, Véronique Taché, Laurel Finta, Daniel Tancredi

**Affiliations:** 1 Department of Pediatrics, University of California Davis Medical Center, 2516 Stockton Blvd, Sacramento, CA 95817 USA; 2 Obstetrics and Gynecology, University of California Davis Medical Center, Sacramento, CA USA

**Keywords:** Delayed cord clamping, Elective cesarean delivery, Safety pilot, Maternal blood loss, Newborn

## Abstract

**Background:**

Delayed cord clamping (DCC) results in decreased iron deficiency in infancy. The American College of Obstetrics and Gynecology has called for research on the optimal time to clamp the cord during cesarean deliveries (CD). Our objective was to conduct a pilot trial examining the safety of delayed cord clamping (DCC) for maternal-infant dyads during elective cesarean delivery (CD).

**Methods:**

We enrolled 39 dyads [23 at 90 s, 16 at 120 s; (DCC Pilot)] between 10/2013 and 9/2014. We abstracted data from the electronic medical record (EMR) for historical controls (HC) birthing between 1/2012–6/2013 for whom DCC was not performed (*n* = 112).

**Results:**

Available data for 37 mothers and 30 infants compared to HC revealed 174 (95% CI: 61–286) mL lower mean estimated maternal blood loss [(EBL) mean (SD) mL]: DCC Pilot 691(218) vs. HC 864(442), *p* = 0.003 and lower incidence of maternal transfusions, DCC Pilot 2.7% vs. HC 18.8%, *p* = 0.016. There was no significant between group difference between DCC Pilot and HC in other a priori definitions of excess maternal blood loss: a) EBL > 800 ml, 21.6% vs. 38.8%, *p* = 0.07 or b) post-op hgb/pre-op hgb < 80%, 16.7% vs. 20.6%, *p* = 0.81. There were also no statistically significant between group differences in rates of NICU admission DCC Pilot 8.1% vs. HC 7.1%, *p* = 1.0., but there was a higher rate of newborn cold stress or hypothermia ≤36.2 °C in study subjects, DCC Pilot 27.0% vs. HC 11.9%, *p* = 0.038.Prevalence of newborn anemia was decreased [DCC pilot 3.3% (1 of 30) vs. HC 40.0% (4 of 10 infants with data), *p* = 0.012. No infants were polycythemic.

**Conclusions:**

These pilot data suggest cord clamping can be delayed to 120 s during elective CD without increased risk of excessive maternal blood loss. More aggressive prevention of infant heat loss may be warranted. A randomized trial to evaluate long-term maternal and infant outcomes is indicated.

**Trial registration:**

Clinical trials.gov, NCT02229162; registered: 1 September, 2014.

## Background

Iron deficiency is globally the most common nutrient deficiency and is the only such deficiency with significant prevalence in industrialized countries [1]. The most recently published analysis of national data reports the prevalence (± SE) of iron deficiency in US children aged 12–23 months of age at 15.1 (± 1.7) % [2] Iron is essential for normal neuronal development and pre-anemic iron deficiency in infants and young children is associated with poorer neurodevelopment [3, 4]. Prevention of iron deficiency is therefore important to optimize development, and doing so at birth with delayed cord clamping (DCC) is an inexpensive, safe and effective option [5–8]. A commentary in Evidence Based Medicine makes the compelling case that our current practice of early cord clamping unnecessarily increases infant risk for iron deficiency and subsequent poorer neurodevelopmental outcomes [9]. A randomized trial in Sweden documented DCC to successfully reduce an already low prevalence of iron deficiency anemia [10].

There is limited evidence, however, regarding outcomes of DCC in cesarean deliveries (CD). No studies to our knowledge have analyzed maternal or infant outcomes for term cesarean deliveries separately from vaginal deliveries. Further, there are different definitions of ‘delayed cord clamping’ with some experts recommending a delay of at least 2 min [5] The American College of Obstetrics and Gynecology identified the timing of umbilical cord clamping after CD vs. vaginal births as an especially important area for future research [11]. They also noted a paucity of data to support or refute the benefit of DCC for term infants in resource rich settings. To begin addressing these gaps, our objective was to perform a pilot safety trial of DCC during cesarean delivery (CD) of term infants to determine if longer intervals (90 and 120 s) relates to poorer maternal and/or newborn outcomes vs. historical controls. Our primary outcome was maternal blood loss and we hypothesized that the time to cord clamping can be safely increased from immediate clamping, to 2 min in CD without causing an increase in adverse outcomes including excessive maternal blood loss, moderate or severe neonatal hypothermia, polycythemia or neonatal ICU admission for respiratory distress. We chose a target of 2 min as the World Health Organization (WHO) recommends a delay of 1–3 min in both vaginal and cesarean deliveries [12] with some experts recommending a minimum of 2 min [5] and it is the time by which most (approximately 55%) of the placental blood has been transfused into the infant.

## Methods

### Study subjects and design

After study approval by the Institutional Review Board at the University of California Davis Medical Center (UCDMC), a convenience sample of dyads undergoing elective CD at term was recruited. Inclusion criteria included women ≥18 years of age with a singleton pregnancy scheduled for an elective CD at ≥37 weeks gestation. Dyads were excluded if mother or infant were medically unstable, or the mother had poorly controlled diabetes mellitus, multiple gestations, or there were known fetal anomalies and/or severe fetal growth restriction. Whenever possible, written consent of the father of the child was obtained in addition to that of the mother.

Operating room procedures included presence of the research assistant in order to alert obstetric, nursing, anesthetic and neonatology staff that the dyad was enrolled in the DCC study. Immediately upon delivery of the infant (at which time the clock began timing delay) and before cord clamp, anesthesiology began administration of IV oxytocin at a rate of 20 mU/min and the infant was dried, placed on the operating table (warmed underneath the sterile drapes by a ‘bear hugger’) between the mothers’ legs and covered with a sterile blanket and hat. The research assistant notified the team when 90 s (or 120 s for the subsequent arm) had elapsed. After cord clamp, cord traction was applied along with manual uterine pressure to express the placenta. If intraoperative blood loss was clinically deemed excessive, or the infant or mother was clinically unstable (e.g. no spontaneous respirations by 10 s) the obstetricians were to clamp the cord immediately upon this assessment and the time to clamp recorded.

Recruitment was continued until there were 15 dyads with complete data at 90 s delay, with an interim analysis and review by the Data Safety and Monitoring Board [(DSMB) consisting of 3 members - a statistician, perinatologist and neonatologist] was performed. After review of interim results in comparison with Historical Controls (HC), the DSMB recommended proceeding with recruitment for the 120 s arm, with modification of the protocol to include: a) a specific time to take the infant’s temperature (which was specified at 15 min of age), rather than the clinical protocol being utilized which specifies the newborn temperature will be taken rectally ‘within 30 min of birth’; b) recording operating room temperature; c) specific protocol for prevention of heat loss. While the drying and covering with dry blanket and hat had been previously employed, the addition of a warm, dry blanket was added.

Historical controls (HC) were collected via de-identified EMR data by the information technologists for the time period from January 2012–July 2013 when the EMR template for infant delivery first included a discrete section for DCC (yes/no and if yes, length of time of delay). During this period, non-immediate clamping was utilized per physician discretion; recorded time of those with delay ranged from 30 to 120 s, but was not always recorded. HC group consisted of those with immediate clamping (*n* = 112). Data were excluded from HC for those without a specified ‘no DCC’.A delay of 30 s or longer was considered DCC per EMR template.

The primary outcome was maternal blood loss measured by the anesthesiologist’s visually estimated blood loss (EBL). Excessive blood loss was a priori defined as 1 or more of the following: a) EBL > 800 mL, (per standard institutional practice at the time); b) > 20% difference between pre- and post-operative hemoglobin levels; c) need for a transfusion; d) need for maternal ICU admission for hemodynamic instability (HC data not available for comparison). Maternal blood loss was also measured quantitatively (QBL), via changing suction canisters after the amniotic fluid was suctioned and calculating wet vs. dry weight of surgical drapes and sponges; these data, however, were not available for comparison in HC data. The QBL measurement was not yet implemented clinically at the time of the study and was therefore protocolized by one of the study’s authors (LF) and performed jointly by the head OR nurse and attending obstetrician. If multiple postoperative hemoglobin levels were performed, that closest to 24 h after birth was utilized.

Newborn outcomes were secondary and included the prevalence of: a) neonatal cold stress or hypothermia (≤36.2 °C) on admission; (36.2 °C was chosen as the cut-off as temperatures at or below this indicates routine screening for hypoglycemia per institutional protocol); b) newborn hemoglobin levels determined by venipuncture at 12 h (0–24) of age; c) incidence of newborn anemia or polycythemia (hgb < 14.5 or > 22.5 g/dL) [13]. Hospital protocol was to measure temperatures rectally, however mothers are encouraged to place their healthy infants skin-to-skin in the operating room, and if the infant was skin-to-skin at the time the temperature was taken, an axillary temperature was recorded.

Other measured outcomes included: a) neonatal ICU admission for respiratory distress; and b) phototherapy treatment during the first 2 weeks of life (birth hospitalization or otherwise), in the absence of evidence of hemolysis, as determined by chart review and a follow-up telephone call at 2 weeks. The latter outcomes could not be compared to the HC group as data on reason for NICU admission or on treatment with phototherapy were not available in the de-identified data. Rates of NICU admission for any cause during the first 5 days were therefore compared. This trial was registered at clinicaltrials.gov, NCT02229162.

### Statistical analysis

We enrolled until there were 15 subjects per group with complete data in order to gather preliminary data and to estimate and compare the means and variances between the groups and with data from historical controls. Data were entered into REDCap [14]. A sample size of 12 has been proposed for pilot studies as appropriate for early phase trials when comparing normally distributed outcomes [15]. We chose 15 per group to achieve suitable precision in estimating means and proportions. For group means that have approximately normal sampling distributions, our sample size permits sufficient precision such that 90% confidence intervals (CI) would have half widths less than 0.5 standard deviations (SD) and that estimates of between-group differences in means would have a 90% CI half width of 0.62 SD. For less frequent binomial outcomes, the exact 90% confidence interval for the true probability of a successful outcome in case all 15 of 15 subjects have a successful outcome would be (0.818, 1.00). If the true probability of success is 0.986 or higher, we would have at least an 80% chance of observing successes in all 15 subjects.

Statistical significance testing was conducted using Fisher Exact Test for binary outcomes and with oneway ANOVA for continuous outcomes in SAS. SAS PROC GLIMMIX with robust variance estimation was used to protect inferences against heteroscedacity (varying variance) and with the HC3 adjustment to protect against small-sample biases. Differences in means/incidence from the reference group (HC without DCC) were estimated with 95% CI, using the same robust variance estimation procedures for differences in means of continuous outcomes and using Agresti-Min unconditional exact 95% CI for risk differences for binary outcomes [16, 17] via SAS PROC FREQ using the RISKDIFF(Method = FMScore) option on the EXACT statement, to base the Agresti-Min 95% CI on the Farrington-Manning score statistic.

Finally, in addition to comparing the DCC pilot vs. the HC groups, we compared outcomes between the 90 s and 120 s DCC pilot groups to evaluate for differences between the two durations of delay.

## Results

### Study subjects

**Fig. 1:**
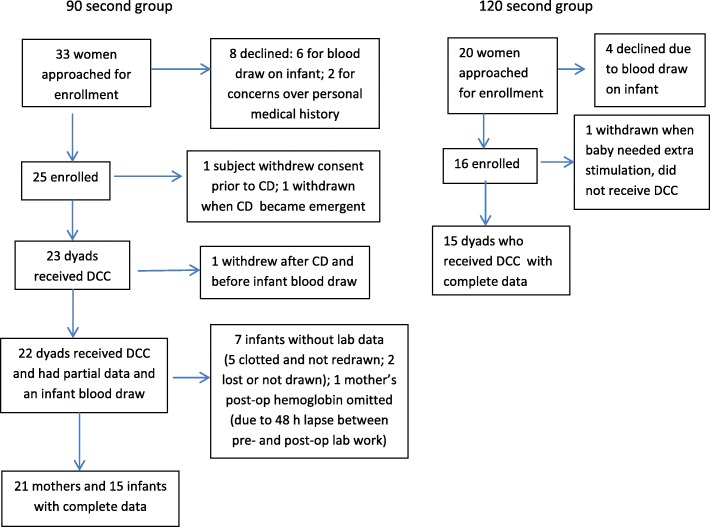
Flow diagram of subject recruitment and enrollment

Study recruitment took place between October 2013 and September 2014 and is diagrammed in Fig. 1. Briefly, a convenience sample of 53 women who met inclusion criteria were approached to participate and a total of 41 consented. Mean (SD) maternal parity and age were 2.7 (1.2) and 32.2 (5.0) respectively.

## DCC pilot vs. historical controls (HC)

### Maternal blood loss

The primary outcome for the mothers was blood loss during surgery (See Table 1). Mean estimated blood loss (EBL) in the DCC pilot group was 174 mL less (95% CI -61, − 286), compared to the HC group, *p* = 0.003. There was also a corresponding lesser percentage of mothers in the DCC group receiving transfusions (2.7%) compared to the HC group (18.8%), *p* = 0.016. There were no statistical between group differences in the other two measures of excess blood loss, EBL > 800 mL or post-operative/pre-operative hgb ratio < 80% (See Table 1).

**Table 1:** Maternal and Newborn Outcomes for Historical Control and DCC Pilot Groups

Maternal Blood Loss –Indicator	DCC Pilot *n* = 39 Mean (SD)	HC n = 112 Mean (SD)	*P* value	Difference in Means/Risk (Risk difference expressed as percentage points) (95%CI)
EBL (mL)	691 (218) *n* = 38	864 (442) *n* = 103	0.003	− 174 (− 286, −81)
Maternal Excess Blood Loss Indicator % yes (n/total)	% yes (n/total)	% yes (n/total)		
EBL > 800 mL	21.6% (8/37)	38.8% (40/103)	0.074	−17.2 (−2.0,32.3)
PostOp/PreOp Hgb < 80%	16.7% (6/36)	20.6% (20/97)	0.81	−4.0 (−17.6, 13.5)
PostOp transfusion	2.7% (1/37)	18.8% (21/112)	0.016	−16.0 (−25.2,-6.9)
Newborn outcomes				
Mean (SD)				
Newborn Hemoglobin (mg/dL)	16.8(2.0) *n* = 30	15.9(3.8) n = 10	0.35	0.8 (−1.8, 3.5)
Admission temperature (°C)	36.6(0.4) *n* = 37	36.7(0.4) *n* = 109	0.08	−0.1 (− 0.3, 0.02)
% yes (n/total)				
Anemia (Hgb < 14.5 g/dL)	3.3% (1/30)	40.0% (4/10)	0.010	−36.7 (−69.2,-5.9)
Cold stress /moderate hypothermia (temp ≤36.2 °C)	27.0% (10/37)	11.9% (13/109)	0.038	15.1 (−0.02,32.6)
NICU admission	8.1% (3/37)	7.1% 8/112	1.0	1.0 (−15.7,7.9)

No mothers in the study required ICU admission for hemodyamic instability.

### Infant outcomes

There were few newborn hemoglobin (hgb) results among the HC group, as hemoglobin was not routinely checked historically. Although the DCC pilot group had a mean hgb 0.9 g higher than the HC control group, this was not statistically significant. There was a lower prevalence of anemia in the DCC pilot vs. HC group, 40.07% vs. 3.3%, *p* = 0.01 (Table 1). No infants in any group were polycythemic.

Mean admission temperatures were not different by group. There were, however, more infants in the DCC pilot group experiencing cold stress or hypothermia (admission temperature ≤ 36.2 °C, 27.0% vs. 11.9%, *p* = 0.038). There was no difference in prevalence of more severe hypothermia of admission temperature < 36.0 °C (data not shown).

Admission to the neonatal ICU occurred in 3 of the DCC pilot group infants (8.1%); all 3 of these were for respiratory distress. We do not have reasons for neonatal ICU admissions in the HC group, but prevalence of ICU admission was nearly identical at 7.1%.

Finally, phototherapy was instituted during the birth hospitalization for only 1 infant that had ABO incompatibility with anti-A antibodies in the DCC pilot group. No infants required readmission for phototherapy. This information was not available for the HC groups.

## DCC 90s vs. DCC 120 s

The only statistically significant difference between the two pilot groups of 90 s (*n* = 23) vs. 120 s (*n* = 16) was in EBL, which was 752 (207) mL for the 90 s group vs. 600 (206) mL for the 120 s group, *p* = 0.04. There was a corresponding greater percentage of mothers in the 90 s group which experienced greater than 20% drop in hgb, (24% vs. 7%), but this was not statistically significant (*p* = 0.16); nor was the greater percentage of mothers in the 90 s group with EBL > 800 mL, 27% vs. 13%, *p* = 0.32.

Interestingly, quantitatively measured blood loss (QBL) in the DCC pilot groups was significantly higher than the EBL; QBL [mean (SD)] was 1056 (507) and 1050 (484) mL for 90 and 120 s vs. EBL of 752 mL (207) and 600 mL (206) respectively, paired t-test *p* = 0.02 and < 0.001. Also, QBL did not corroborate the finding of statistically significantly greater EBL in the 90 vs. 120 s DCC group. Quantitatively measured blood loss was not available in historical controls.

There were no statistically significant differences in any of the newborn outcomes between the 90 and 120 s DCC groups.

## Discussion

The primary outcome results of this pilot safety trial are reassuring. Maternal blood loss, using multiple measurement techniques, was not significantly increased from a clinical or statistical standpoint, in the DCC pilot group compared to the HC group. In fact, two of the measures -EBL and percent of mothers requiring transfusion- actually indicated *lower* blood loss in the DCC Pilot vs. the HC group. These findings support our hypothesis that cord clamping can be safely increased to 2 min in CD without increased maternal blood loss. Similar findings were observed in the 3-arm trial in Argentina [18] (immediate vs. 1 or 3 min of delay), in which 28–30% of deliveries in each arm were CD with no increased blood loss in delayed vs. immediate clamping; results of CD were not analyzed separately however. Maternal blood loss data were separately reported for CD recently when comparing before vs. after instituting a policy of 30 s delay in cord clamp for premature infants; there was no significant difference in EBL during CD for mothers after policy institution [19]. It is interesting to note they also reported a trend towards *less* of a decrease in hemoglobin in those with clamping at 30 s compared to immediate clamping [mean (95% CI) difference 0.4 (0.0,0.08) gm/dL, *p* = 0.05].

The finding of fewer mothers requiring transfusion in the DCC pilot vs. HC group is important to note and suggests mothers undergoing elective CD may actually benefit from DCC. It is unclear if this was related to greater care of the surgeons to prevent blood loss in the setting of late cord clamping (e.g. more clamping off of small bleeding vessels), altered response of the uterus to an emptier placenta or receipt of oxytocin during the delay in the 120 s group, or a combination thereof. It is also possible that this could be a chance finding. The Cochrane review did not find a difference in risk of severe postpartum hemorrhage or need for transfusion by use of prophylactic uterotonic before vs. after clamping, [7] but few studies reported timing of use, and those that did typically used intramuscular administration, likely to have less impact than intravenous administration used in our protocol.

We also note that the quantitatively measured blood loss (QBL) in both DCC pilot groups was significantly greater than that estimated by EBL, by 40 and 75% respectively. This discrepancy is even greater than the 30% noted in the literature previously [20] and supports current recommendations to implement quantitative blood loss measures [21–24]. We acknowledge that the difference in EBL between the 90 and 120 s study groups was not corroborated by the QBL. This raises the concern of possible bias in EBL estimates. It may also reflect implementation challenges with accurate measurement of QBL, as this procedure had not yet been implemented clinically at our institution at the time of the study.

Newborn outcomes of anemia, polycythemia, cold stress/hypothermia, and NICU admission were secondary outcomes; of these only the cold stress/hypothermia measure was statistically significantly increased in the DCC pilot vs. HC groups. While it was reassuring that there was not a difference in prevalence of admission temperature < 36 °C,*.* the rate of admission temperature ≤ 36.2 °C (27%) is nevertheless of concern and may be clinically significant. It is possible that other factors may have contributed to hypothermia, e.g. recent practice of placing the infant skin-to-skin in the operating room per maternal preference. In this setting mothers may also be cool and it may be difficult to cover the infant well. Regardless, we believe more aggressive measures to prevent heat loss are warranted, such as use of polyethylene wraps for the infant during delayed clamping, previously documented to decrease hypothermia in premature infants during resuscitation [25]. A recent institutional protocol for DCC in premature infants < 34 weeks successfully utilized polyethylene wraps along with delivery room temperatures of 76–79 °C to prevent hypothermia in both vaginal as well as cesarean deliveries [19].

No infants in any group were polycythemic, and statistically fewer in the intervention groups were anemic, as might be expected. However, a significant limitation of this study is relatively few infants in the HC group had hemoglobin data available for comparison, and therefore those with data may not represent the overall historical term, elective CD group.

Limitations to this study are several and include this being a small, single-center pilot study, which limits generalizability. Our controls are historical, so results may be confounded by temporal trends and they were not matched for maternal characteristics. Also limiting is that we are unable to confirm if the EMR record of DCC in the historical controls is accurate. As noted, there were limitations in the de-identified newborn data we obtained, with few hemoglobin results, and no data available on treatment with phototherapy or cause for NICU admission. Strengths of the study include observation of the time to cord clamp, and objective measures of maternal blood loss in addition to EBL.

Despite the above limitations, our findings suggest that cord clamping can be safely delayed for at least 120 s in elective CD without resulting in excess maternal blood loss and further suggest maternal blood loss may be *decreased* with the protocol utilized. Given the potential benefit of improved iron status on infant neurodevelopmental outcomes and the dearth of data available in this country regarding infant iron status and related outcomes, these results call for a larger, randomized trial of DCC in elective CD evaluating both short- and longer-term outcomes for both members of the dyad. The need for such a trial is particularly compelling with the recent report that delayed cord clamping in Sweden, a high-income country, reduced the number of children with low fine-motor and social skill scores at 4 years of age [26]. An editorial in the same JAMA issue highlighted that since 2000, no randomized trial has documented symptomatic polycythemia in infants [27]. Intuitively, there is reason to think newborn outcomes related to DCC are likely similar to those in vaginal deliveries, but this needs to be confirmed prior to recommending its use. Lastly, it will be important to confirm if this 120 s delay can also improve outcomes for the mother, an intriguing possibility which is suggested by our and other data.

## Conclusions

Data from this pilot safety study suggest that delaying cord clamping for 2 min in elective, term cesarean deliveries does not increase risk of excessive maternal blood loss. More aggressive prevention of infant heat loss than utilized in our protocol may be warranted. A randomized trial to evaluate longer term maternal and infant outcomes is indicated.

## Abbreviations

C: Centigrade
CD: cesarean delivery
CI: confidence interval
DCC: delayed cord clamping
DSMB: Data Safety and Monitoring Board
EBL: estimated blood loss
EMR: electronic medical record
gm/dL: grams/deciliter
h: hour
HC: historical control
hgb: hemoglobin
ICU: intensive care unit
IQR: interquartile range
IV: intravenous
min: minutes
mL: milliliter
mU: milliunits
OR: operating room
QBL: quantitatively measured blood loss
s: seconds
SD: standard deviation
